# Application of PolyHIPE Membrane with Tricaprylmethylammonium Chloride for Cr(VI) Ion Separation: Parameters and Mechanism of Transport Relating to the Pore Structure

**DOI:** 10.3390/membranes8010011

**Published:** 2018-03-02

**Authors:** Jyh-Herng Chen, Thi Tuyet Mai Le, Kai-Chung Hsu

**Affiliations:** 1 Department of Materials and Mineral Resources Engineering, National Taipei University of Technology, 1, Section 3, Chung-Hsiao East Road, Taipei 10608, Taiwan; 2 College of Engineering, National Taipei University of Technology, 1, Section 3, Chung-Hsiao East Road, Taipei 10608, Taiwan; tuyetmaimt@gmail.com (T.T.M.); kaichung0226@gmail.com (K.-C.H.)

**Keywords:** PolyHIPE, pore structure, Cr(VI), diffusion coefficient, transport study

## Abstract

The structural characteristics of membrane support directly affect the performance of carrier facilitated transport membrane. A highly porous PolyHIPE impregnated with Aliquat 336 is proposed for Cr(VI) separation. PolyHIPE consisting of poly(styrene-*co*-2-ethylhexyl acrylate) copolymer crosslinked with divinylbenzene has the pore structure characteristic of large pore spaces interconnected with small window throats. The unique pore structure provides the membrane with high flux and stability. The experimental results indicate that the effective diffusion coefficient D* of Cr(VI) through Aliquat 336/PolyHIPE membrane is as high as 1.75 × 10^−11^ m^2^ s^−1^. Transport study shows that the diffusion of Cr(VI) through Aliquat 336/PolyHIPE membrane can be attributed to the jumping transport mechanism. The hydraulic stability experiment shows that the membrane is quite stable, with recovery rates remaining at 95%, even after 10 consecutive cycles of operation. The separation study demonstrates the potential application of this new type of membrane for Cr(VI) recovery.

## 1. Introduction

The supported liquid membrane (SLM) technique is a promising liquid membrane technique which allows extraction/separation/removal of metal ions for various wastewater treatments [1,2,3,4,5,6]. The advantages of SLMs over traditional separation technologies are the low inventory of the organic phase used, lower capital and operating costs, and low energy consumption for mass transfer by combining extraction and stripping in one single stage. Furthermore, with proper selection of impregnated extractant, one can relatively easily modify the SLM with selectivity toward the target metal ions [7,8]. 

The most important factors that influence the performance of supported liquid membrane (SLM) system are physical stability of the impregnated carrier and the rate of the mass transfer through the membrane. The structural characteristics of membrane support directly affect the performance of carrier facilitated transport membrane [9]. Many studies have focused on the search of new membrane support. The strategies for the developments of new membrane support included creating dense interface gel layers inside the pores [10], coating with surface layer [11], functionalization of membrane support [12], formation of composite membranes [13,14] and improvement of pore structure of membrane [15]. A suitable membrane support should have pore structure characteristics of high porosity, pore size distribution, and proper tortuosity [16]. 

The porous PolyHIPE materials have high porosity provided by large amounts of spherical voids highly connected with small window throats [17]. With high porosity, PolyHIPE can incorporate substantial amounts of metal ion extractant into the membrane for metal ion extraction. In addition, a porous membrane with high interconnectivity can have high transport efficiency [18]. Furthermore, the small window throats may also be responsible for higher extractant stability, due to the capillary effect. Therefore, in our previous study we have demonstrated that PolyHIPE with highly interconnected macroporous structure can be advantageous for membrane applications [15]. The remarkable combination of high flux and high stability of Aliquat 336/PolyHIPE membrane has prompted us to further investigate the parameters and mechanisms of carrier facilitated transport relating to the pore structure of PolyHIPE membrane.

Facilitated transport through membranes is a complex and coupled process. It is important to understand and quantify the coupling to describe the transport in membranes. To understand the facilitated transport of ions through PolyHIPE membrane, Cr(VI) transport through PolyHIPE membrane impregnated with Aliquat 336 is used as a model system because of the following reasons. Cr(VI) is very important in many industry applications, such as electroplating, leather tanning, and industrial metal alloys. Cr(VI) is toxic compound, and hazardous to the environment. Therefore, the study of recovery of Cr(VI) is interesting from both environment and resource recycling points of view. Furthermore, numerous studies of Cr(VI) separation provide abundant comparisons with the system in this study. 

It was a concept common to many models that aim at relating macroscopic transport properties of porous materials to their microstructure [19]. In this study, the pore structure of PolyHIPE substrate was characterized based on the pore size distribution, porosity, tortuosity, and constrictivity, to evaluate membrane properties. The transport behavior of Cr(VI) was examined in order to elucidate the mechanism for the facilitated transport of Cr(VI) ions through Aliquat 336/PolyHIPE membrane. The feasibility for practical application of the PolyHIPE membrane was demonstrated by the long-term performance of the membrane. 

## 2. Experimental

### 2.1. Chemicals

Tricaprylmethylammonium chloride (Aliquat 336, CH_3_N[(CH_2_)_7_CH_3_]_3_Cl) was obtained from Alfa Aesar. Potassium chromate (K_2_Cr_2_O_7_) was purchased from Kanto Chemical (Tokyo, Japan). The chromium standard for atom adsorption analysis was obtained from J.T. Baker. Styrene (St, C₆H₅CH=CH₂), divinylbenzene (DVB, C_6_H_4_(C_2_H_3_)_2_), 2-ethylhexyl acrylate (EHA, CH_2_=CHCOOC_8_H_17_), sorbitan monooleate (Span 80, C_24_H_44_O_6_), azobisisobutyronitrile (AIBN, C_8_H_12_N_4_), sodium hydroxide (NaOH), and hydrochloric acid (HCl) were obtained from Aldrich (Milwaukee, WI, USA). All chemicals were of reagent grade, and used without further purification.

### 2.2. Preparation and Characterization of PolyHIPE Membrane

In a typical experiment, the continuous phase was organic solution consisting of St (3.0 g), EHA (6.0 g), DVB (1.0 g), AIBN (0.2 g), and Span 80 (2.4 g). The dispersed phase (40.0 mL of DI-water) was then added dropwise (within 30 min) to the organic solution, stirring at 300 rpm. After the addition of water, the mixture was stirred at 400 rpm for another 30 min to obtain a viscous emulsion solution for membrane preparation. The emulsion solution (HIPE) was placed in a mold with two flat polytetrafluroethylene (PTFE) plates, and circumscribed by spacers. The thickness of membrane was controlled at 120 ± 5 µm. The mold was then tightly clamped and put into oven pre-set at 60 °C to start polymerization. After 48 h of polymerization, the PolyHIPE membrane was carefully retrieved. The PolyHIPE membrane was washed three times in ethanol solution for 6 h to remove organic impurities, followed by immersion in water for 1 h to remove inorganic residual. Finally, the PolyHIPE membrane was oven-dried at 50 °C for 24 h. 

The bulk density was determined by dividing the mass of sample with the bulk volume, as determined from the dimensions of the sample. The membrane dimension was measured by a digital micrometer (Mitutoyo 293-100, Mitutoyo, Aurora, IL, USA) with 0.1 µm resolution over 10 readings. The chemical structure of PolyHIPE membrane was characterized by FT-IR spectrometer (NICOLET 6700, Thermo Electron, Waltham, MA, USA). The microstructure morphology was examined by SEM (S-4700, Hitachi, Chiyoda-Ku, Tokyo, Japan). The characteristic of pore structure of PolyHIPE was evaluated by mercury porosimetry (Micromeritics AUTOPORE 9520, Micromeritics Instrument, Norcross, GA, USA) with maximum test pressure of 200 MPa.

The tensile testing of the PolyHIPE membrane was conducted according to ASTM D412. The Shore hardness of the PolyHIPE membrane was measured with SHAHE/LX-C 0-100 HC (Wenzhou Sanhe Measuring Instrument, Wenzhou, China). The flexibility of PolyHIPE was evaluated by following the standard test method protocol of ASTM F137-08. By placing the specimen over a mandrel with different radius, the bent position of the specimen face was visually examined for breaks, cracks, or other damage. The testing was performed in an ambient environment within 5 min. 

### 2.3. Preparation of Aliquat 336/PolyHIPE Membrane 

The PolyHIPE membrane was immersed in 50 mL of ethanol containing different amounts of Aliquat 336. The impregnation was carried out at room temperature for 12 h. After vacuum dried at room temperature, the amount of impregnated Aliquat 336 was determined by the weight difference before and after the impregnation process with the average of three samples. 

### 2.4. Cr(VI) Transport Study 

The membrane transport experiments were performed in a double-cell device with solution volume of 120 mL for each compartment. The contact area of the Aliquat 336/PolyHIPE membrane was 16 cm^2^. The feeding phase was a Cr(VI) aqueous solution with varied initial pH adjusted with HCl. The stripping phase is a basic solution of NaOH. Separation experiments were conducted at room temperature (25 ± 0.1 °C). The concentration of Cr(VI) in both phases was measured with an Atomic Absorption spectrometer (Analyst 100, Perkin-Elmer, Waltham, MA, USA) with a chromium cathode lamp (Perkin Elmer, Waltham, MA, USA) at a wavelength of 357.9 nm. All data are averages of three replicable determinations. In this study, separation experiments were performed for feeding solution with initial pH of 2, 3, 4, and 5 to simulate the pH of Cr(VI) wastewaters from electroplating plant [20]. The stripping phase was set to be pH 12, at which an optimal stripping efficiency can be obtained as determined in the pre-experiment. In this study, the prepared supported liquid membrane was conditioned for 12 h in the cell with pure water. After this induction period of time, the Cr(VI) was introduced to begin the transport study.

The removal and recovery rate of Cr(VI) was defined as Equations (1) and (2):

(1) $$Removal(\%)=\frac{{\left[C\right]}_{fo}-{\left[C\right]}_{f}}{{\left[C\right]}_{fo}}\times 100$$

(2) $$Recovery(\%)=\frac{{\left[C\right]}_{s}}{{\left[C\right]}_{fo}}\times 100$$

where [*C*]*_fo_* is the initial concentration of Cr(VI) in the feeding phase, [*C*]*_f_* and [*C*]*_s_* is concentration of Cr(VI) in the feeding and stripping phase at time *t*, respectively.

## 3. Results and Discussion

### 3.1. Chemical and Physical Characteristics of PolyHIPE Membrane 

The characteristics of the PolyHIPE membrane depend on the composition of monomers, crosslinking agent, organic/aqueous phase ratio, and the amount of surfactant used for the preparation of emulsion solution (HIPEs). In order to prepare a feasible membrane substrate, the PolyHIPEs with different monomer compositions were prepared and evaluated (Table 1). In this study, the monomers were styrene (St) and 2-ethylhexylacrylate (EHA). The use of EHA was to improve the flexibility of the substrate. The amount of crosslinking agent, DVB, was fixed at 10 wt % of organic phase, as suggested by a previous study [21]. The amount of Span 80 was 20 wt % of the organic phase, and the volume ratio of organic and aqueous phase was 2:8. All the compositions were pre-tested to have good emulsion stability during the preparation of PolyHIPE. 

**Figure 1:**
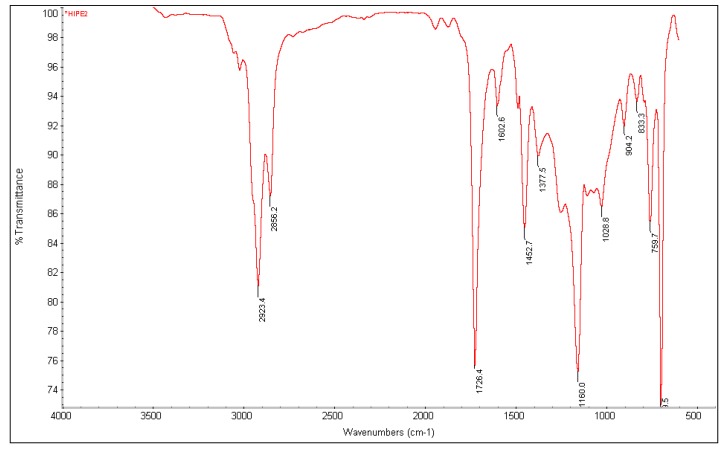
FT-IR spectrum of PolyHIPE membrane (30 wt % styrene (St), 60 wt % 2-ethylhexylacrylate (EHA), and 10 wt % divinylbenzene (DVB)).

Figure 1 shows the FTIR of PolyHIPE. The characteristic peaks at about 700; 1453; 2856; and 2923 cm^−1^ are associated with the methylene group of styrene [22]. The stretching vibration of carbonyl group (C=O) of EHA is seen at 1726 cm^−1^. The peak at 1603 cm^−1^ is due to the skeletal deformation vibration of C–H in DVB [23]. Therefore, the PolyHIPE used in this study is a poly(styrene-*co*-2-ethylhexyl acrylate) copolymer crosslinked with DVB.

The physical property of PolyHIPE substrate is affected by the styrene/EHA/DVB ratio. Table 1 shows that the hardness of membrane increases almost linearly with increasing styrene fraction. For styrene fractions higher than 60 wt %, the PolyHIPE substrates are glassy and brittle, which are not suitable to be used as a membrane. For EHA fraction of 90 wt %, the PolyHIPE substrate is very soft and too sticky to handle. With 90 wt % of styrene, the porosity is about 0.68 ± 0.02, which is much smaller than that of initial volume fraction of aqueous phase (0.8) in the emulsion solution. This may be due to shrinkage of poly(styrene-*co*-2-ethylhexylacrylate) after polymerization. The specific volume of substrate decreased with increasing styrene content. The porosity for EHA fraction of 60 wt % and 90 wt % is 0.77 ± 0.03 and 0.78 ± 0.01, respectively, which are very close to 0.80, indicating that EHA can reduce the shrinkage of PolyHIPE substrate. 

In the followed study, the PolyHIPE membrane was prepared with 20 wt % organic phase (30 wt % St, 60 wt % EHA, and 10 wt % DVB) and 80 wt % aqueous phase. The averaged Young’s modulus and Shore hardness of the substrate is about 1.25 ± 0.42 MPa and 52.6 ± 4.5 LX-C, respectively. The flexibility evaluated according to (ASTMF 137-08) shows that the porous PolyHIPE can be bent over a cylindrical mandrel with diameter as small as 0.2 cm. With this composition, a strong and elastomeric membrane can be robustly obtained. The mechanical properties of PolyHIPE membrane are acceptable for long term separation operation, as demonstrated in the reusability study in this study (Section 3.2.3).

### 3.2. Pore Structure of PolyHIPE Membrane 

For treatment process optimization and design purposes, it is important to relate the process macroscopic behavior, with regard to flow and mass transfer, to the pore structure of the porous material [24]. In this study, two characterization methods, mercury porosimetry and image analysis were adopted to obtain information about the porous structure of PolyHIPE membrane. Mercury porosimetry measurement can provide pore size distribution of membrane. Image analysis of membrane cross sections can reveal the pore geometry and structure, which can be used to determine tortuosity and constrictivity of the membrane. This information can be used to correlate the transport behavior with the pore structure characteristics of the PolyHIPE membrane.

#### 3.2.1. Pore Size Distribution

**Figure 2:**
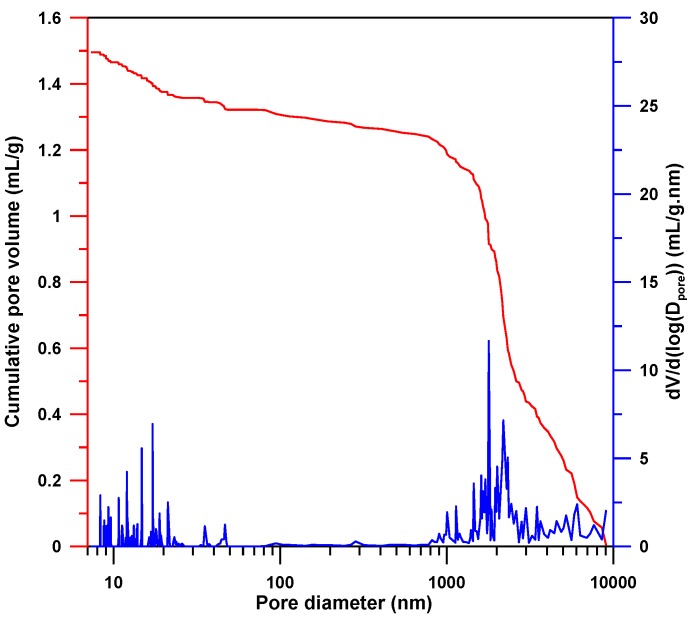
Pore diameter distribution of PolyHIPE membrane (30 wt % St, 60 wt % EHA, and 10 wt % DVB).

Figure 2 shows the cumulative volume with respect to pore diameter measured with mercury porosimetry. The data of cumulative volume shows a two-step intrusion behavior. The first step at 1000 to 9000 nm describes the mercury intrusion into large pores of PolyHIPE, while the second step with slope change below 30 nm is for the small pores. Figure 2 also shows the log differential intrusion volume (d*V*/d(log(*D_pore_*)) with respect to pore diameter, where *V* is the cumulative volume of mercury and *D_pore_* is the pore diameter. The pore size of PolyHIPE membrane shows a bimodal distribution with two distinct pore diameter regions (10–30 nm and 1000–9000 nm) with a “valley” within 30–1000 nm. The relative pore volume of the two distinct pore size ranges is 11.6% (10–30 nm) and 88.4% (1000–9000 nm), respectively. Other characteristic properties of PolyHIPE membrane obtained from mercury porosimetry measurement are bulk density (0.23 g/mL), skeletal density (1.095 g/mL), and porosity (0.77).

#### 3.2.2. Tortuosity 

**Figure 3:**
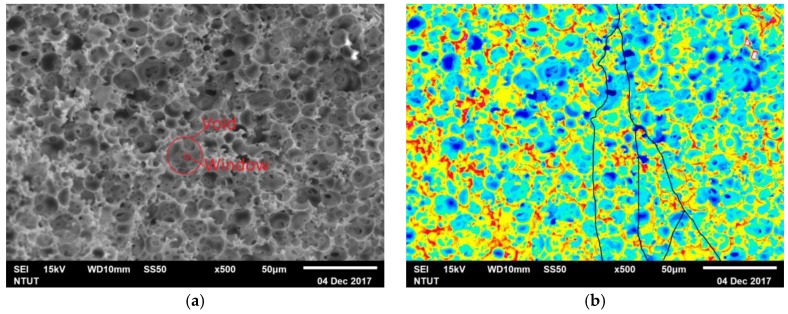
(**a**) SEM of PolyHIPE membrane (30 wt % St, 60 wt % EHA, and 10 wt % DVB) cross section. (**b**) Binarized SEM image by built-in SEM program, with black lines to indicate the *L_actual_*.

Tortuosity (*τ*) accounts for the increase in the distance of a diffusing ion due to bending and branching of pores, which affects the effective diffusivity of ions in the PolyHIPE membrane. The tortuosity is usually defined as the ratio of the actual path length through the pores to the Euclidean distance [25]:

(3) $$\tau =\frac{L_{\mathrm{actual}}}{L_{\mathrm{Euclidean}}}$$

where *L_actual_* is the actual path length through the pores, and *L_Euclidean_* is the shortest distance between the start and end points in Euclidean space. The tortuosity of porous material can be estimated based on an SEM image analysis process [26,27]. In this study, the values of *L_actual_* and *L_Euclidean_* of PolyHIPE membrane was determined from the SEM images (Figure 3a), of which the images can be processed by using both Adobe Photoshop CS5 and the built-in SEM program with several binarization parameter settings to improve the accuracy and eliminate uncertainties of path length determination (Figure 3b). However, the SEM images show partly the inner structure of the samples [28]. In order to obtain acceptable tortuosity measurement, the SEM image was taken from various sections of PolyHIPE membrane, and by taking five visually identifiable routes of each binarized image the tortuosity can be estimated (Figure 3b). The measured tortuosity of PolyHIPE is within the range of 1.08–1.21. 

Alternatively, *τ* can be also estimated by a number of theoretical models from porosity [29]. For a porosity Φ higher than 0.5, the tortuosity can be estimated by using the equation *τ* = 0.8(1 − Φ) + 1 [30]. In this study, since the porosity of PolyHIPE membrane is about 0.77, the calculated tortuosity is equal to 1.18, which lies within the range of tortuosity estimated by SEM image analysis. The low tortuosity is apparently due to the high porosity of PolyHIPE.

#### 3.2.3. Constrictivity

The constrictivity (*β*) is defined as the ratio of *A_min_*/*A_max_* = πr_min_^2^/πr_max_^2^, where *A_min_* is the pore cross section area at the constriction, and *A_max_* is the cross section at nonconstricted “bulges” in the pore [31]. Since the constrictivity is defined not for a single pore, but as the parameter of the entire pore space considered, several methods have been suggested to estimate the constrictive factor of porous material [32]. In this study, the constrictivity is statistically estimated directly from the SEM observation of the diameters of void and window throat. In order to obtain acceptable constrictivity measurement, the SEM images were taken from various sections of PolyHIPE membrane and the diameter of five visually identifiable pores and throats were determined from each SEM image (Figure 3a). The averaged pore diameter is 10 ± 2.5 µm, and the averaged throat diameter is 0.6 ± 0.2 µm. Therefore, the estimated constrictivity is about 3 × 10^−3^ ± 0.3 × 10^−3^. The diameter of pore and window throat is close to the range of 1000–9000 nm as determined by the mercury porosimetry.

### 3.3. Preparation of Aliquat 336/PolyHIPE Membrane

**Figure 4:**
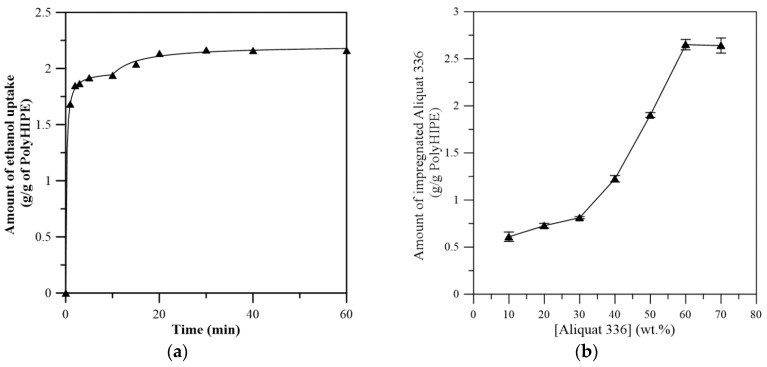
(**a**) Absorption of ethanol by PolyHIPE. (**b**) Effect of Aliquat 336 concentration in ethanol on the mount of immobilized Aliquat 336.

Due to the high viscosity of Aliquat 336, the impregnation of the extractant requires the aid of suitable solvent. In this study, ethanol was selected as the solvent, since ethanol is a good solvent for extractant. Solvent absorption in PolyHIPE is important, since it governs the subsequent extractant impregnation and quality of the final membrane. Figure 4a shows the uptake curve of ethanol in PolyHIPE. The curve exhibits a high initial uptake rate followed by a slower absorption of solvent in the later stage. The pattern of solvent uptake in PolyHIPE membrane suggests a possible two-step process. For each stage of solvent uptake, the shape of the weight gain curves resembles that of other accumulation phenomena with an asymptotic equilibrium [33]. Therefore, the kinetic of solvent uptake behavior can be described by two-stage Peleg model (Equation (4)) [33,34].

(4) $$R(t)=\frac{\frac{1}{K_{11}}t}{1+\frac{K_{21}}{K_{11}}t}+\frac{\frac{1}{K_{12}}\left[\left(t-t_{b}\right)+\left|t-t_{b}\right|\right]}{2+\frac{K_{22}}{K_{12}}\left[\left(t-t_{b}\right)+\left|t-t_{b}\right|\right]}$$

where *R(t)* is the weight ratio (g of solvent uptaken/g of PolyHIPE), *t_b_* is the break point time of associated two-stage curve, *K_ij_* stands for the related constant (*i* = 1 for the Peleg rate constant and *i* = 2 for the Peleg capacity constant), *j* for different stages of absorption.

The fitted parameters of two-step Peleg model show that the solvent uptake rate of the first stage (1/*K*_11_ = 11.765) is significantly higher than that of the second stage (1/*K*_12_ = 0.043). The higher initial uptake rate in the first stage is due to the fast diffusion of ethanol into the large cavities of PolyHIPE, while the second stage of absorption is attributed to the slower diffusion of ethanol into the small cavities. The amount of ethanol uptake in the first and second stage of absorption is 1.94 and 0.22 g/g PolyHIPE, respectively. It is interesting to note that the amount of ethanol uptake in the second stage of absorption is about 11.6% of the total amount, corresponding to the relative pore volume of small pore size as determined by mercury porosimetry. 

For the impregnation of Aliquat 336, the amount of immobilized Aliquat 336 increases with increasing Aliquat 336 concentration in ethanol (Figure 4b). At about 60 vol % of Aliquat 336, the highest amount of impregnated Aliquat 336 in PolyHIPE membrane is 2.64 ± 0.10 g/g of PolyHIPE membrane, indicating that the PolyHIPE is filled with Aliquat 336. 

### 3.4. Transport Study of Cr(VI) through Aliquat 336/PolyHIPE Membrane

To demonstrate the feasibility of PolyHIPE membrane, transport of Cr(VI) through Aliquat 336/PolyHIPE membrane was investigated as a model system. The transport of Cr(VI) by the carrier across the support liquid membrane is known to involve five consecutive steps [35].

(1) Diffusion of Cr(VI) from feeding phase to the interface.
(2) Formation of Cr(VI)–extractant complex at the feed phase–membrane interface.
(3) Diffusion of Cr(VI)–extractant complex through the membrane.
(4) Dissociation of Cr(VI)–extractant complex at the stripping phase–membrane interface, to release the Cr(VI) into the stripping phase.
(5) Diffusion of the Cr(VI) from the interface to the stripping phase.

Steps (1) and (5) are related to the mixing condition of the bulk phase, which can be presumed to be a fast process, if both bulk phases are effectively stirred. For Steps (2) and (4), the rate is related to the formation and dissociation of the complex at the interfaces. For Step (3), the characteristics of membrane pore structure is the critical factor affecting the diffusion of Cr(VI)–extractant complex through the membrane.

#### 3.4.1. Influence of the Stirring Rate

**Figure 5:**
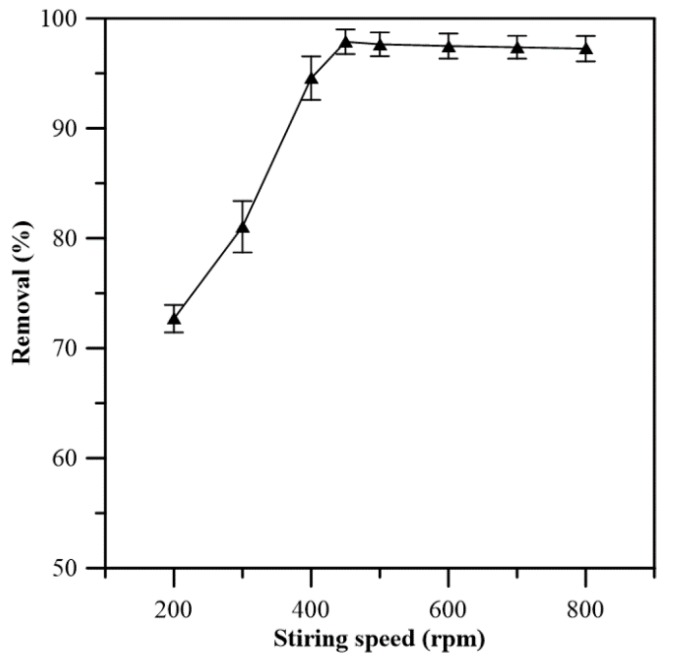
Influence of the stirring speed on the removal rate. Feeding solution: [Cr(VI)] = 53 mg/L at pH 4; stripping phase: NaOH solution at pH 12.

The influence of stirring rate on the removal rate of Cr(VI) was studied in order to obtain a suitable stirring operation condition. Figure 5 shows that the Cr(VI) removal rate increases with increasing stirring rate. In the range of 450–500 rpm, the removal rate reaches a maximum of about 98% after 8 h of separation, indicating that the diffusion of Cr(VI) between the bulk phase and membrane interface (Steps 1 and 5) is minimized. The results of stirring rate higher than 500 rpm show only very slight decrease of the averaged removal rate. Nevertheless, the decrease is within experimental error. Therefore, the stirring rate for feeding and stripping phase was fixed at 450 rpm throughout the followed transport study.

#### 3.4.2. pH Effect on the Transport of Cr(VI) through the Aliquat 336/PolyHIPE Membrane

**Figure 6:**
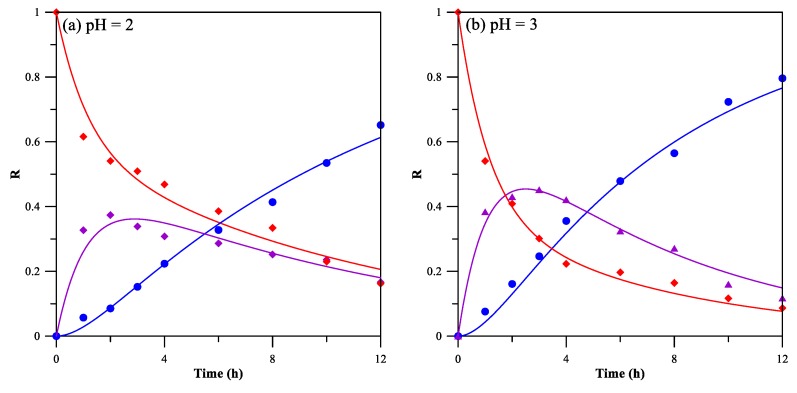

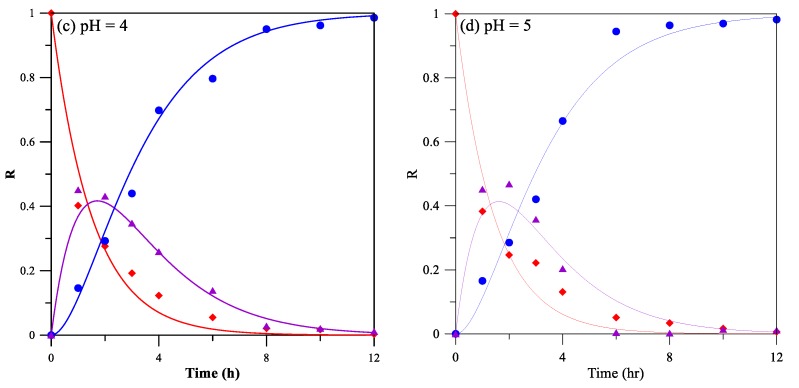
The variations of Cr(VI) concentrations with respect to time. *R_f_* (♦), *R_m_* (▲), and *R_s_* (●). Feeding phase: [Cr(VI)] = 53 mg/L; stripping phase: NaOH solution at pH 12; stirring rate: 450 rpm.

Figure 6 shows the variations of Cr(VI) concentrations with respect to time. The pH of feeding phase was varied from 2 to 5. The pH of the stripping phase was kept at 12, at which an optimal stripping efficiency can be obtained, as determined in the pre-experiment. Assuming both the extraction and stripping of Cr(VI) follow the first order reaction with “apparent rate constants” as a function of extraction reaction, stripping reaction, and the solute diffusion rate [36], the variation of Cr(VI) concentration in feeding, membrane, and stripping phases can be described by a kinetic law of two consecutive first order irreversible reaction (5) [15,37].

(5) $${\mathrm{Cr}(\mathrm{VI})}_{f}\overset{k_{1}}{\underset{}{\to }}{\mathrm{Cr}(\mathrm{VI})}_{m}\overset{k_{2}}{\to }{\mathrm{Cr}(\mathrm{VI})}_{s}$$

where *Cr*(*VI*)*_f_*, *Cr*(*VI*)*_m_*, and *Cr*(*VI*)*_s_* represent the Cr(VI) ions in the feeding, membrane, and stripping phase, respectively. *k*_1_ and *k*_2_ are the first order apparent rate constants of the extraction and the stripping reaction, respectively. 

The variation of Cr(VI) concentration can be expressed by the following differential Equations (6)–(8):

(6) $$\frac{dR_{f}}{dt}=-\alpha k_{1}R_{f}$$

(7) $$\frac{dR_{m}}{dt}=\alpha k_{1}R_{f}-\beta k_{2}R_{m}$$

(8) $$\frac{dR_{s}}{dt}=\beta k_{2}R_{m}$$

where *α* and *β* is the ratio of membrane contact area to the volume of the feeding and stripping phase, respectively (*α* = *β* = 13.3 m^2^/m^3^ in this study). *R_f_*, *R_m_*, and *R_s_* are the dimensionless concentrations of Cr(VI) (with respect to [*C*]*_fo_*) in feeding, membrane, and stripping phase, respectively. 

Table 2 shows the fitted apparent rate constants. The apparent extraction rate constant *k*_1_ first increases approximately linearly as the feeding phase pH increases from 2 to 4, and then levels off at pH 5, indicating that the extraction of Cr(VI) at lower pH is a relatively slower process than that at higher pH. The apparent stripping rate constant *k*_2_ shows similar trend, despite the fact that the stripping phase is of the same pH (pH 12) for all the transport study. The change of *k*_2_ may be due to the difference between stripped Cr(VI)–Aliquat 336 complex formed at different feeding phase pH. 

The initial feeding flux can be used to quantify the transport efficiency of Cr(VI) through the PolyHIPE membrane. At the initial instant of transport, the flux *J_f_*^0^ based on the concentration in the feeding phase can be described by Equation (9). The derivative d[*C*]*_f_*/d*t* can be estimated from the fitted equation of Equation (6), provided that the fitted equation has high correlation coefficient (*R*^2^ > 0.95).

(9) $$J_{f}{}^{0}=-{\frac{V}{S}\frac{dC_{f}}{dt}|}_{t=0}=-\frac{V}{S}k_{1}C_{fo}$$

where *S* is the operation membrane area (cm^2^), *V* is the volume of the feeding phase (cm^3^). 

Table 2 also shows the initial flux of Cr(VI) transport through Aliquat 336/PolyHIPE membrane at different feeding phase pH. The low initial flux at pH 2 may be due to that the formation of chromate aggregate complex in the membrane phase. The complexed Cr(VI) may form condensation products of chromate aggregate as (R_3_CH_3_N^+^)_2_Cr_3_O_10_^2−^ and/or (R_3_CH_3_N^+^)_2_Cr_4_O_13_^2−^ [38]. Then, the *J_f_*^0^ increases with increasing pH, and levels off at pH 4. Since within the pH range of this study, the Cr(VI) species distribution is almost the same (as determined by Mineql +4.6), the extraction step in this study can be expressed as following reactions:

(10) $$R_{3}{\mathrm{CH}}_{3}N^{+}{+\mathrm{HCrO}}_{4}{}^{-}\to \left(R_{3}{\mathrm{CH}}_{3}N^{+}\right){\mathrm{HCrO}}_{4}{}^{-}$$

(11) $$2\left(R_{3}{\mathrm{CH}}_{3}N^{+}\right){\mathrm{HCrO}}_{4}{}^{-}\to {\left(R_{3}{\mathrm{CH}}_{3}N^{+}\right)}_{2}{\mathrm{Cr}}_{2}O_{7}{}^{2-}$$

(12) $$2\left(R_{3}{\mathrm{CH}}_{3}N^{+}\right){+\mathrm{Cr}}_{2}O_{7}{}^{2-}\to {\left(R_{3}{\mathrm{CH}}_{3}N^{+}\right)}_{2}{\mathrm{Cr}}_{2}O_{7}{}^{2-}$$

(13) $${\left(R_{3}{\mathrm{CH}}_{3}N^{+}\right)}_{2}{\mathrm{Cr}}_{2}O_{7}{}^{2-}{+\mathrm{HCrO}}_{4}^{-}{+H}^{+}↔{\left(R_{3}{\mathrm{CH}}_{3}N^{+}\right)}_{2}{\mathrm{Cr}}_{3}O_{10}{}^{2-}{+H}_{2}O$$

(14) $${\left(R_{3}{\mathrm{CH}}_{3}N^{+}\right)}_{2}{\mathrm{Cr}}_{2}O_{7}{}^{2-}{+2\mathrm{HCrO}}_{4}^{-}{+2H}^{+}↔{\left(R_{3}{\mathrm{CH}}_{3}N^{+}\right)}_{2}{\mathrm{Cr}}_{4}O_{13}{}^{2-}{+2H}_{2}O$$

Based on the initial flux, the transport efficiency of Cr(VI) through Aliquat 336/PolyHIPE membrane is comparable to other studies (Table 3). It is also worth pointing out that even for trace concentrations of Cr(VI), the residual amount of Cr(VI) is less than 0.5 ppm after 10 h of separation, meeting the environmental regulation standard of Taiwan. 

#### 3.4.3. Modeling and Parameters of Cr(VI) Diffusion

Since the aforementioned “apparent rate constant” is a function of Cr(VI)–Aliquat 336 complex reaction and the solute diffusion rate, determination of the Cr(VI)–Aliquat 336 formation constant and diffusion coefficient is required in order to elucidate the transport behavior of Cr(VI) through PolyHIPE membrane. In this study, since the bulk phase is effectively stirred, i.e., when the aqueous diffusion layer is compressed to a minimum, the film diffusion of Cr(VI) between bulk phase and membrane interface is a fast process, aqueous analytical concentrations [*C*]*_f_* and [*C*]_s_ are uniform throughout the feeding and stripping phases, respectively, and the concentration of Cr(VI) complex changes linearly inside the membrane [45,46,47]. Assuming that the rate-determining step is the diffusion of Cr(VI) through the membrane, the formation and dissociation of the Cr(VI)–Aliquat 336 complex at the interfaces are fast processes [35]. The effective diffusivity (D*) is key, and the most convenient parameter to describe the process of diffusion through porous membrane, since the value of D* is directly related to the pore structure characteristics of PolyHIPE membrane due to the tortuosity and constrictivity effects. 

The determination of effective diffusion coefficient can generally be achieved from the relationship observed between the flux and the concentrations of diffusant in the feeding phase [46]. From Fick’s first law, the diffusion flux inside the membrane (*J_m_*) can be defined as Equation (15).

(15) $$J_{m}=D^{*}\frac{d\left[AC\right]}{dl}=D^{*}\frac{{\left[AC\right]}_{f}-{\left[AC\right]}_{s}}{l}$$

where D* is the effective diffusion coefficient of Cr(VI)–Aliquat 336 complex inside the membrane, [*AC*] is the concentration of Cr(VI)–Aliquat 336 complex in the membrane phase, subscripts *f* and *s* refer to the feeding and stripping interfaces of the membrane, and *l* is the membrane thickness. Since the stripping phase is at very high pH (pH 12), the dissociation of Cr(VI)–Aliquat 336 is a fast process, and the concentration of complex [*AC*]*_S_* is practically nil. On the other hand, the formation of complex is a fast heterogeneous equilibrium [37]. Therefore, the concentration of complex [*AC*]*_f_* can be related to the concentration of Cr(VI) in the feeding phase according to the mass action law, Equation (16):

(16) $${\left[AC\right]}_{f}=K\left[A\right]\;{\left[C\right]}_{f}$$

where *K* is the formation constant of the Cr(VI)–Aliquat 336 complex, [*A*] and [*C*]*_f_* are the concentrations of the Aliquat 336 in the membrane phase and the concentration of Cr(VI) in the feeding phase, respectively. The total carrier concentration [*A*]*_o_* immobilized in the membrane is constant, equal to the sum of the concentrations [*A*] and [*AC*]*_f_* (Equation (17)). Therefore, the concentration of complex [*AC*]*_f_* can be related to the concentration of Cr(VI) in the feeding phase by Equation (18).

(17) $${\left[A\right]}_{o}=\left[A\right]+{\left[AC\right]}_{f}$$

(18) $${\left[AC\right]}_{f}=\frac{{\left[A\right]}_{o}\times K\times {\left[C\right]}_{f}}{1+K\times {\left[C\right]}_{f}}$$

Since the formation of Cr(VI) complex is a fast process, based on the quasi-steady state assumption *J_m_*^0^ ≈ *J_f_*^0^, the initial flux can be described as

(19) $$J_{f}{}^{0}=\left(\frac{D^{*}}{l}\right)\frac{\left({\left[A\right]}_{o}K{\left[C\right]}_{fo}^{}\right)}{1+K{\left[C\right]}_{fo}^{}}$$

The postulated mechanism indicates that *J_f_*^0^ is related to the effective diffusion coefficient and formation constant of Cr(VI)–Aliquat 336 complex. In order to test the proposed relationship, Equation (19) was rearranged to a linear form, as in Equation (20). 

(20) $$\frac{1}{J_{f}{}^{0}}=\left(\frac{l}{D^{*}}\right)\left(\frac{1}{{\left[A\right]}_{0}K}\right)\left(\frac{1}{{\left[C\right]}_{fo}^{}}\right)+\left(\frac{l}{D^{*}}\right)\left(\frac{1}{{\left[A\right]}_{0}}\right)$$

Transport experiments were carried out with [*C*]*_fo_* which varied in the range 0.2 × 10^−3^–4 × 10^−3^ M. By plotting the values of 1/*J_f_*^0^ vs. 1/[*C*]*_fo_*, the effective diffusion coefficient and formation constant of the complexes can be obtained from the slope and intercept, respectively. 

(21) $$D^{*}=\frac{l}{{\left[\bar{A}\right]}_{o}\times \mathrm{intercept}}$$

(22) $$K=\frac{\mathrm{intercept}}{\mathrm{slope}}$$

**Figure 7:**
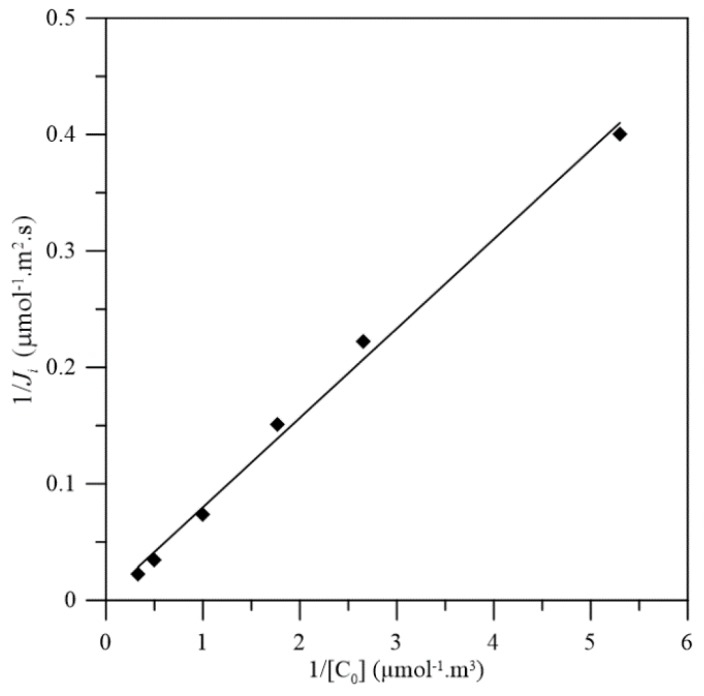
Plots of 1/*J_f_*^0^ vs. 1/[*C*]*_fo_*, for the transport of Cr(VI) across the Aliquat 336/PolyHIPE membrane. Feeding phase: pH 4; stripping phase: pH 12. Stirring rate: 450 rpm.

Figure 7 shows that the plot of 1/*J_f_*^0^ vs. 1/[*C*]*_fo_*, is linear with high correlation coefficient (*R*^2^ = 0.99). Hence, the experimental results are in agreement with a mechanism in which the diffusion of the complex is the rate-determining step. Moreover, it confirms that the complex involved in the transport process has 1:1 stoichiometry as the HCrO_4_^−^–Aliquat 336 complex characterized as Equation (10), since under the solution condition, over 90% of Cr(VI) species is HCrO_4_^−^.

From the slope and intercept of the above result (Figure 7), the effective diffusion coefficient D* is about 1.75 × 10^−11^ m^2^ s^−1^, and the formation constant K is about 5.68 mol^−1^ L. By substituting the porosity (0.77), constrictive factor (3.3 × 10^−3^), and tortuosity (1.18) into the equation D* = *DβΦ/τ*, we can obtain the free bulk diffusion coefficient of Cr(VI)–complex (*D*) equal to 8.14 × 10^−9^ m^2^ s^−1^. 

For comparison, the free bulk diffusion coefficient for the Cr(VI)–Aliquat 336 complex was also estimated using Wilke–Chang equation (Equation (23)) [45].

(23) $$D=7.4\times 10^{-8}{\left(\phi M\right)}^{1/2}\frac{T}{\eta V_{}^{0.6}{}_{}}$$

where *M* and *η* is the molecular weight and viscosity of solvent, and *φ* is the “association parameter” (*φ* = 1). Since the PolyHIPE is filled with Aliquat 336, which can be treated as solvent in this case, *M* = 404.16 g/mol and *η* = 1.5 Pa.s. The value of *V* = 0.671 × 10^3^ cm^3^/mol can be calculated by summing the contribution of atoms, which consist of the solute molecule (i.e., Cr(VI)–Aliquat 336 complex) as suggested by Wilke–Chang [46]. For 25 °C and *T* = 298 K, *D* = 5.95 × 10^−13^ m^2^ s^−1^. 

The value of *D* obtained from D* seems to be very high, compared to that estimated from Wilke–Chang equation (Equation (23)). It suggests that the migration of the complex is not a pure diffusion process. One possible explanation is the jumping transport mechanism as suggested by many literature studies [48]. With this mechanism, the transporter molecules act as “stepping stones”, and the solute moves through the membrane by jumping from one fixed site to another. The characteristic of jumping transport is to be faster than the diffusion [35]. Therefore, for the conditions of the experiments, the assumption of jumping transport seems to be correct. 

#### 3.4.4. Stability of Aliquat 336/PolyHIPE Membrane

One major concern of using Aliquat 336/PolyHIPE membrane is the stability of impregnated extractant. The stability of the Aliquat 336/PolyHIPE was investigated by immersing the membrane in water under magnetic stirring at 450 rpm. Experimental results show that during the first 20 min period of immersion, the amount of impregnated Aliquat 336 in PolyHIPE membrane decreases about 5%. This may be due to the removal of loosely adhered Aliquat 336 from the surface of membrane, since Aliquat 336 is slightly soluble in water. After 60 min, the amount of impregnated Aliquat 336 becomes stable. It is worth to note that during the stability test, the amount of Aliquat 336 removed into the water phase is about 0.00125 g/100 mL, which is much smaller than the solubility of Aliquat 336 in aqueous phase (about 0.1 g/100 mL) [49]. This result indicates that the pore structure of PolyHIPE can effectively improve the stability of Aliquat 336 inside the membrane. 

**Figure 8:**
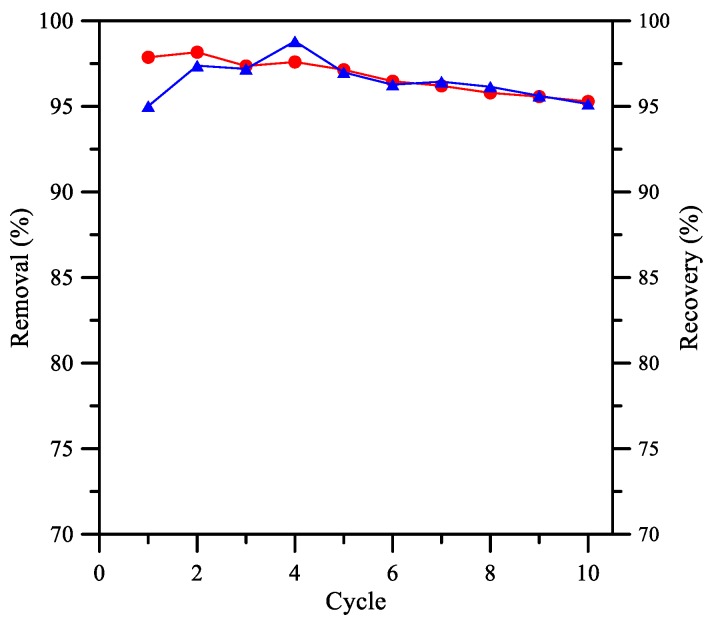
Removal and recovery rates in consecutive cycle: (●) removal rate, (▲) recovery rate. Feeding phase: [Cr(VI)] = 53 mg/L at pH 4; stripping phase: pH 12; stirring rate: 450 rpm.

The hydraulic stability of Aliquat 336/PolyHIPE membrane was evaluated based on the removal and recovery rate of Cr(VI), to demonstrate the reusability of PolyHIPE membrane for the separation of Cr(VI). Figure 8 shows that after ten sequential cycles of 8 h separation of Cr(VI), the removal rate drops slightly from 97.9% to 95.3%, while the recovery rate remained approximately 95% after 10 cycles. 

### 3.5. Separation of Cr(VI) from Mixed Ion Solution

Industrial wastewaters contain mixtures of heavy metal ions and other anionic species. In this study, the Aliquat 336/PolyHIPE membrane was used for the recovery of Cr(VI) from a simulated wastewater mixture. The composition of the mixture solution was based on a wastewater from local (Taichung, Taiwan) electroplating plant: Cr(VI) (53.2 mg/L), Ni(II) (65.0 mg/L), Mg(II) (81.8 mg/L), Cu(II) (42.4 mg/L), SO_4_^2−^ (245.0 mg/L), and F^−^ (84.30 mg/L), at pH 4.0. 

Table 4 shows the results of separation. The recovery rate of Cr(VI) is as high as 95.09%, even under the interference of high concentrations of SO_4_^2−^ and F^−^. The separation factor of Cr(VI)/SO_4_^2−^ and Cr(VI)/F^−^ is 27.19 and 11.17, respectively. The high separation factor demonstrates that Aliquat 336/PolyHIPE membrane has potential for practical application in the recovery of Cr(VI) from industrial wastewater.

## 4. Conclusions

A highly porous PolyHIPE membrane prepared with 20 wt % organic phase (30 wt % St, 60 wt % EHA, and 10 wt % DVB) and 80 wt % aqueous phase has structural characteristics as the membrane support for supported liquid membrane. The PolyHIPE membrane has a bimodal pore size distribution with two distinct pore diameter regions (10–30 nm and 1000–9000 nm). The relative pore volume of the two distinct pore size ranges is 11.6% and 88.4%, respectively. The tortuosity of PolyHIPE is within the range of 1.08–1.21. Due to the high porosity (0.77) and low tortuosity, the Aliquat 336–PolyHIPE membrane shows high flux for Cr(VI) separation. The effective diffusion coefficient of Cr(VI) is as high as 1.75 × 10^−11^ m^2^ s^−1^. Based on the characteristic parameters of pore structure, transport study shows that the diffusion of Cr(VI) through Aliquat 336/PolyHIPE membrane can be attributed to the jumping transport mechanism. The hydraulic stability study shows that the membrane is quite stable with recovery rate remain 95% even after 10 consecutive cycles. The separation study demonstrates the potential of practical application of this new type of membrane for Cr(VI) recovery.

## Figures and Tables

**Table 1:** HIPE compositions and quality of resulting PolyHIPE membrane.

St:EHA:DVB ^a^	Visual Physical Quality	Shore Hardness LX-C	Porosity (%)	Specific Volume (cm^3^/g)
90:0:10	hard, brittle	85.0 ± 2.5	0.68 ± 0.02	3.22 ± 0.28
60:30:10	hard, brittle	65.1 ± 3.4	0.71 ± 0.01	3.70 ± 0.57
30:60:10	flexible, strong	52.6 ± 4.5	0.77 ± 0.03	4.35 ± 0.35
0:90:10	elastomeric, sticky	32.5 ± 6.2	0.78 ± 0.01	4.76 ± 0.59

^a^ Monomer mixture composition expressed as wt %. Styrene (St); 2-ethylhexylacrylate (EHA); divinylbenzene (DVB).

**Table 2:** The fitted apparent rate constant and initial flux in the feeding phase.

pH	*k*_1_ (×10^−6^ m/s)	*k*_2_ (×10^−6^ m/s)	*R*^2^			*J_f_*^0^ (µmol/m^2^ s)
			Feeding	Membrane	Stripping	
2	8.33	3.96	0.99	0.95	0.99	8.54
3	12.11	4.38	0.99	0.98	0.99	12.13
4	13.54	9.58	0.98	0.96	0.99	14.38
5	14.58	9.79	0.97	0.98	0.99	14.52

Note: Feeding solution: [Cr(VI)] = 53 mg/L; stripping phase: NaOH solution at pH 12; stirring rate: 450 rpm.

**Table 3:** Comparison of the Cr(VI) transport efficiency with different membranes.

[Cr(VI)]_o_	Feeding Phase	Membrane Type/Base Polymer	Carrier	*J_f_*^0^	Membrane Thickness	Ref.
(mol/L)	pH			(µmol/m^2^ s)	(µm)	
2.0 × 10^−4^	0.12	PIM/CTA	Calix[4]arene	2.253	42	[39]
2.0 × 10^−3^	1	PIM/CTA	Aliquat 336	8.84	28	[40]
2.3 × 10^−4^	1.2	PIM/CTA	Aliquat 336	3.11	80	[41]
1.8 × 10^−6^	8	PIM/CTA	Aliquat 336	0.002	62	[42]
1.0 × 10^−3^	1	ACM	Cyanex 923	10.91	90	[14]
1.0 × 10^−3^	1	ACM	Cyanex 923	11.94	25	
3 × 10^−4^	2	SLM	CYPHOS IL101	5.5	125	[43]
7.5 × 10^−4^	0	SLM	Cyanex 921	6.7	125	[44]
1.0 × 10^−3^	4	SLM/PolyHIPE	Aliquat 336	14.38	120	this study

Note: polymer inclusion membrane (PIM), cellulose triacetate (CTA), activated composite membrane (ACM).

**Table 4:** Separation of Cr(VI) from mixture solution by Aliquat 336/PolyHIPE membrane.

Ion Matter	Recovery (%)	Separation Factor ^(a)^
Cr(VI)	95.09	1
Cation		
Ni(II)	1.23	77.07
Mg(II)	0.78	120.60
Cu(II)	1.61	86.75
Anion		
SO_4_^2−^	3.49	27.19
F^−^	8.51	11.17

^(a)^ yAyBxAxB, where *y_A_* and *y_B_* are the concentration of components *A* and *B* in the stripping phase; *x_A_* and *x_B_* are the concentrations of the components in the feeding phase [50].
